# Widespread Occurrence of *Bd* in French Guiana, South America

**DOI:** 10.1371/journal.pone.0125128

**Published:** 2015-04-22

**Authors:** Elodie A. Courtois, Philippe Gaucher, Jérôme Chave, Dirk S. Schmeller

**Affiliations:** 1 Station d'Ecologie Expérimentale du Centre National de la Recherche Scientifique (CNRS) à Moulis, Unité de Service et de Recherche (USR) 2936, Saint Girons, France; 2 Centre National de la Recherche Scientifique Guyane, Unité de Service et de Recherche (USR) 3456, Cayenne, Guyane Française; 3 Laboratoire Ecologie et Diversité Biologique, Unité Mixte de Recherche (UMR) 5174, Toulouse, France; 4 Helmholtz-Centre for Environmental Research, Department of Conservation Biology, Leipzig, Germany; 5 Université de Toulouse, Laboratoire Ecologie Fonctionnelle et Environnement (Ecolab), Toulouse, France; 6 Centre National de la Recherche Scientifique, EcoLab, Toulouse, France; Imperial College Faculty of Medicine, UNITED KINGDOM

## Abstract

The amphibian chytrid fungus *Batrachochytrium dendrobatidis* (*Bd*) is a purported agent of decline and extinction of many amphibian populations worldwide. Its occurrence remains poorly documented in many tropical regions, including the Guiana Shield, despite the area’s high amphibian diversity. We conducted a comprehensive assessment of *Bd* in French Guiana in order to (1) determine its geographical distribution, (2) test variation of *Bd* prevalence among species in French Guiana and compare it to earlier reported values in other South American anuran species (http://www.bd-maps.net; 123 species from 15 genera) to define sentinel species for future work, (3) track changes in prevalence through time and (4) determine if *Bd* presence had a negative effect on one selected species. We tested the presence of *Bd* in 14 species at 11 sites for a total of 1053 samples (306 in 2009 and 747 in 2012). At least one *Bd*-positive individual was found at eight out of 11 sites, suggesting a wide distribution of *Bd* in French Guiana. The pathogen was not uniformly distributed among the studied amphibian hosts, with Dendrobatidae species displaying the highest prevalence (12.4%) as compared to Bufonidae (2.6 %) and Hylidae (1.5%). In contrast to earlier reported values, we found highest prevalence for three Dendrobatidae species and two of them displayed an increase in *Bd* prevalence from 2009 to 2012. Those three species might be the sentinel species of choice for French Guiana. For *Dendrobates tinctorius*, of key conservation value in the Guiana Shield, smaller female individuals were more likely to be infected, suggesting either that frogs can outgrow their chytrid infections or that the disease induces developmental stress limiting growth. Generally, our study supports the idea that *Bd* is more widespread than previously thought and occurs at remote places in the lowland forest of the Guiana shield.

## Introduction

Over a third of extant amphibian species are currently considered at an elevated threat of extinction [1–3]. The causes of these declines are multiple, including climate change, habitat destruction, and emerging infectious diseases [4]. In pristine amphibian rich areas, especially in Central America, amphibian declines have been associated with outbreaks of the chytrid fungus *Batrachochytrium dendrobatidis* (*Bd*), the agent of chytridiomycosis [5,6]. *Bd* has been shown to infect over 500 species of the 7000 known amphibian species and it occurs on all continents except Antarctica [7]. Its current and historical distribution, however, remains poorly documented in many parts of the world, especially in the Neotropics where sampling is limited due to difficult logistics (see www.bd-maps.net, [8]).

*Bd* has locally been reported in Ecuador [9], Uruguay [10], Venezuela [11], Peru [12], Argentina [13], Chile [14], Colombia [15] and coastal Brazil [16] and therefore in nearly the entirety of South America. Despite these numerous studies, we currently lack a comprehensive understanding of *Bd* distribution in South America. In the remote and isolated Venezuelan tepuis, unusual mortality events were reported between 1984 and 1986, but no evidence of *Bd* infection was found in 37 museum specimens collected during those years [11]. Previous sampling for *Bd* in Suriname [17] and a recent study of *Bd* in French Guiana caecilians [18] have yielded negative results, but in 2009, the presence of *Bd* has been unambiguously confirmed from two sites in French Guiana [19].

The prevalence of *Bd* in the tropics has long been believed to be limited to mid and high elevations [20,21]. Indeed, temperature appears to be an important factor of variation in *Bd* virulence as optimal temperatures of *Bd* growth range from 17 to 25°C [22] and the prevalence and intensity of *Bd* infections tend to be greater during cooler months of the year [23]. However, records of *Bd* in lowland tropical forest amphibian populations (between 50 and 100 m a.s.l.) has increased in past years [15,16,24,25] and recent studies tend to show that the pathogen may exhibit local adaptation to temperatures above 25°C, typical of lowland tropical forests. Indeed, isolates coming from different locations display distinct upper thermal maxima for growth [26]. Documenting the distribution of *Bd* in Neotropical lowland forests should help assessing the level of risk to the South American amphibian fauna.

A puzzling aspect of the emergence of amphibian chytridiomycosis has been that even if epizootics have been observed at many locations, some amphibian communities are currently coexisting alongside *Bd* with no evidence of pathogenic effects of this disease [27]. This can be due to the existence of different *Bd* strains, differing in their virulence (from hypovirulent lineages to the hypervirulent *Bd*-GPL strain [27]), or to species-specific susceptibility to *Bd* [28]. The extent to which evolutionary relationships among host species determine their susceptibility of infection remains understudied (but see [28]). Variation in *Bd* prevalence among species may also be explained by differences in life history traits and especially breeding strategies [7,29]. Species that are more dependent on water are expected to be the most impacted species [30] due to prolonged larval stages and therewith prolonged exposure to *Bd* zoospores and due to *Bd* zoospores apparently being highly susceptible to desiccation [31].

Here, we report on a systematic *Bd* assessment in the tropical forest of French Guiana, an amphibian biodiversity hotspot harboring 110 amphibian species [32] and an additional 20 candidate species [33], most of which being endemic to the Guiana shield. The mean temperature of French Guiana is 27°C, slightly higher than *Bd* maximum growth temperature of 25°C [22] but lower than temperatures reported lethal to *Bd* [34]. The desiccation risk of *Bd* might be limited in French Guiana, as annual rainfall ranges between 1650 and 4000 mm, with an increasing rainfall towards the East. Our goals were to (1) determine the spatial distribution of *Bd* in this country, (2) identify the variation of prevalence among the species, compare it to values reported in the literature and seek ‘sentinel’ species to improve future disease surveillance, (3) identify the variation of *Bd* prevalence through time for a selected set of species and (4) test whether *Bd*-infection has an impact on the body condition index and health condition of one flagship species (*Dendrobates tinctorius*). This focus on *D*. *tinctorius* is also of interest as this species is widely distributed in Brazil, French Guiana, Guyana, and Suriname, is subject to legal and illegal trade world-wide [35], is widely displayed in zoos around the world, and might be a reservoir for *Bd* [36]

## Materials and Methods

### Study sites and sampled species

In February-March 2009, five sites (Favard, Inselberg, Pararé, Trésor and Matoury) were visited to swab anuran species in an opportunistic approach to sample for *Bd* (N = 306 individuals). Originally, we targeted four abundant species belonging to two amphibian families, Dendrobatidae (*Dendrobates tinctorius*—N = 197, *Allobates femoralis*—N = 10) and Bufonidae (*Rhinella margaritifera*—N = 49, and *Atelopus flavescens*—N = 50). Of these, data for two sites and one species (*D*. *tinctorius*) were reported earlier [19]. In September 2011, we sampled one site Trinité followed by a more extensive sampling from January to March 2012. We swabbed individuals at the same five sites and an additional six sites, for a total of 11 sites (Table 1; N = 747 individuals). We opportunistically sampled 14 focal species, including five Dendrobatidae (*Dendrobates tinctorius*—N = 215, *Allobates femoralis*—N = 30, *Anomaloglossus baeobatrachus*—N = 99, *Anomaloglossus aff*. *degranvillei*—N = 20 *and Ranitomeya amazonica*—N = 15), four Bufonidae (*Rhinella margaritifera*—N = 104, *Rhinella castaneotica*—N = 53, *Atelopus flavescens*—N = 96 and *Amazophrynella sp*.—N = 16), four Hylidae (*Dendropsophus minutus*—N = 30, *Dendropsophus leucophyllatus*—N = 17, *Hypsiboas punctatus*—N = 10 and *Scinax boesemani*—N = 12) and one Microhylidae (*Chiasmocleis shudikarensis*—N = 30) (Table 2). In 2012, all sites except one (Trinité) were therefore sampled within a time period of three months (January to March) corresponding to the beginning of the rainy season (amphibian breeding season) in French Guiana [37].

**Table 1:** Location of the11 sampled sites, locality, and mean elevation.

Sites	2009 sampling	GPS coordinates	Mean elevation (a.s.l.)	Number of samples in 2011–2012
ADNG	No	N 05°33'49.0" W 53°56'41.4"	30	79
**Trinité**	**No**	**N 04°37'22.0'' W 53°17'16.1''**	**120**	**31**
Saint Elie	No	N 05°17'59.0'' W 53°03'05.1''	70	55
Montagne des singes	No	N 05°04'30.3'' W 52°41'19.1''	50	56
**Matoury**	**Yes**	**N 04°52'15.0" W 52°21'10.0"**	**100**	**30**
**Favard**	**Yes**	**N 04°30'18.0" W 52°02'45.0"**	**150**	**50**
**Trésor**	**Yes**	**N 04°36'26.0'' W 52°16'46.0''**	**150**	**70**
**Inselberg**	**Yes**	**N 04°04'05.7'' W 52°41'22.1''**	**200**	**99**
**Pararé**	**Yes**	**N 04°02'43.0'' W 52°40'49.0''**	**100**	**175**
Saut Maripa	No	N 03°48'38.8'' W 51°53'46.0''	50	78
Saint-Georges	No	N 03°59'13.5'' W 51°53'24.6''	90	24

Sites located within protected areas are highlighted in bold.

**Table 2:** Description of the species sampled in 2011–2012.

Family	Species	Adult habitat	Larvae Habitat
Dendrobatidae	*Dendrobates tinctorius* (Cuvier, 1797)	terrestrial	phytotelms
	*Allobates femoralis* (Boulenger, 1884)	terrestrial	phytotelms
	*Anomaloglossus baeobatracchus* (Boistel and Massary 1999)	terrestrial	direct development
	*Anomaloglossus aff*. *degranvillei*	riparian	direct development
	*Ranitomeya amazonica* (Schulte, 1999)	terrestrial	phytotelms
Bufonidae	*Rhinella margaritifera* (Laurenti, 1768)	terrestrial	pools and streams
	*Rhinella castaneotica* (Caldwell 1991)	terrestrial	pools and streams
	*Atelopus flavescens* (Duméril and Bibron 1841)	terrestrial	stream
	*Amazophrynella sp*.	terrestrial	ponds
Hylidae	*Dendropsophus minutus* (Peters, 1872)	arboreal	ponds
	*Dendropsophus leucophyllatus* (Beireis, 1783)	arboreal	ponds
	*Hypsiboas punctatus* (Schneider, 1799)	arboreal	ponds
	*Scinax boesemani* (Goin, 1966)	arboreal	ponds
Microhylidae	*Chiasmocleis shudikarensis* (Dunn, 1949)	terrestrial	ponds

The presence of *Bd* was assayed using sterile swabs (MW 100–100, Medical Wire and Equipment, Bath) by rubbing feet, belly, and legs of each sampled individual. Swabs were stored in the shadow immediately after sampling and permanently stored at 4°C as soon as possible. Cross contamination was avoided by using sterile latex gloves that were changed for each individual.

For each sampled individual of *D*. *tinctorius*, we also measured the SVL (Snout Vent Length) using a caliper (precision 0.1 mm), and the body weight (precision 0.01g). We computed the Body Condition Index (BCI) for each *D*. *tinctorius* individual as the residuals of the regression of the SVL against the cubic root of the weight. The BCI is an accurate proxy for lipid content in amphibians and a good indicator of the individual health status [38]. After manipulation, the sampled frogs were released at the point of capture.

Amphibian species protection status in French Guiana required no specific authorizations for such a capture-release protocol (individuals were caught, rubbed with the sterile swab, and released at the point of capture). For sites located within protected areas (Table 1 in bold), permissions to conduct the capture-release protocol were obtained from the administrators of the reserves (Réserve de la Trinité for the site Trinité, Réserve des Marais de Kaw-Roura for site Favard, Réserve du Mont Grand Matoury for the site Matoury, Réserve Trésor for site Trésor and Réserve des Nouragues for sites Pararé and Inselberg). Permits covered the non-invasive swabbing procedure that we performed on the amphibians, not demanding any special considerations by an Animal ethic committee. Stress due to capture and handling were kept to the necessary minimum, with immediate release of each individual at the site of capture.

### Molecular analyses

DNA was extracted from swabs using a PrepMan extraction [39]. Briefly, 60 μl of PrepMan Ultra (Applied Biosystems) were added to each sample (tip of the swab) along with 30 to 40 mg of silica beads (0.5 mm diameter, Biospec Products) in a 1.5 mL Eppendorf tube. The sample was then homogenized for 45 s in a Mini Beadbeater 16 (Biospec Products) and centrifuged for 30 s at 14000 rpm. These steps were repeated twice, followed by a heating period of 10 min at 96°C. Tubes were then cooled for 2 min and centrifuged at 14000 rpm for 3 min. A volume of 20–40 μL of supernatant was recovered and used to test for *Bd* presence. Aliquots of DNA were permanently stored at -20°C.

Presence of *Bd* in the sample was tested with a quantitative real-time PCR taqman assay [39] run in doublet. Each plate included internal positive controls (Internal Positive Control Reagents, Applied Biosystems) to detect amplification inhibition. Each 96-well assay plate also included standards of known *Bd* quantity of strain IA043, kindly provided by Matthew Fisher (control samples contained DNA from 100, 10, 1 and 0.1 *Bd* genome equivalents—GE) and negative controls with no DNA template. These standards were used to construct a quantification curve to determine the *Bd*-load of each sample [39,40]. To prevent inhibition by the extraction reagent, extractions were diluted by a factor of ten in distilled water prior to the PCR [39]. A sample was defined as *Bd*-positive when both replicates were positive and when the *Bd* load was greater than 0.1 GE considering the dilution factor of the sample, a threshold also considered as acceptable in other studies [41,42]. Thirty-seven samples found to remain single positive or double positive with a GE < 0.1 even after reruns, were considered as *Bd* negative (S1 Table). Mean prevalence per species and per family were computed by dividing the total number of positive individuals by the total number of sampled individuals.

### Meta-analysis

We compared our results with a compilation of literature values as reported in the *Bd*-map database (http://www.bd-maps.net, [8]). We extracted records and prevalence of *Bd* for wild specimens from Central and South America in genera occurring in French Guiana. We obtained prevalence data for 123 species representing 15 genera.

### Statistical analyses

We tested whether the Dendrobatidae species sampled here displayed higher *Bd* prevalence compared to species belonging to other genera (Bufonidae, Hylidae or Microhylidae) using a non-parametric Wilcoxon test. Difference in *Bd* load among families was tested using a non-parametric Kruskal-Wallis test. We tested whether prevalence significantly differed between 2009 and 2011–2012 using Fisher's exact test. For *D*. *tinctorius*, differences in SVL, weight and BCI among infected and uninfected males and females (N = 117 males and 83 females) were tested using a non-parametric Wilcoxon test.

## Results

**Fig 1:**
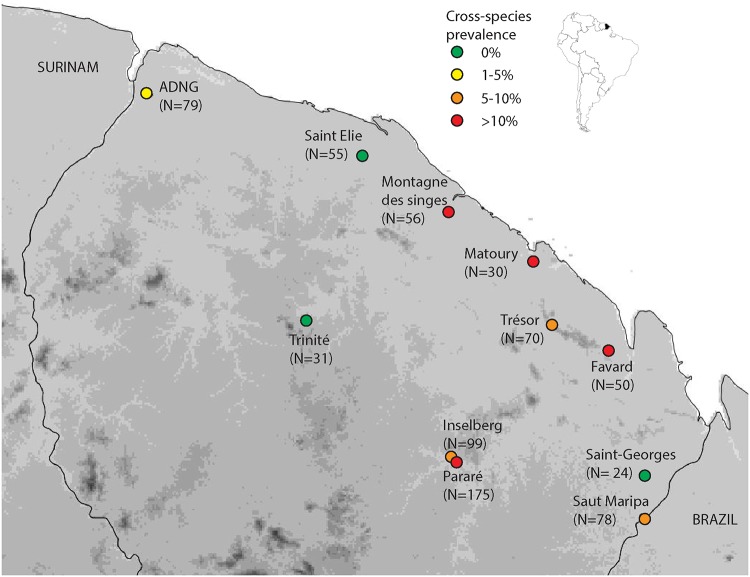
Location of the 11 sites surveyed in 2011–2012. *Bd*-negative sites are indicated with green circles and *Bd*-positive sites are indicated either in yellow (1–5% cross species prevalence), orange (5–10% cross species prevalence) or in red (>10% cross species prevalence).

In 2011–2012, eight of the 11 study sites were found to contain at least one *Bd*-positive individual (Fig 1). *Bd*-positive sites were distributed at elevations ranging from 30 to 200 m a.s.l. (Table 1).

**Fig 2:**
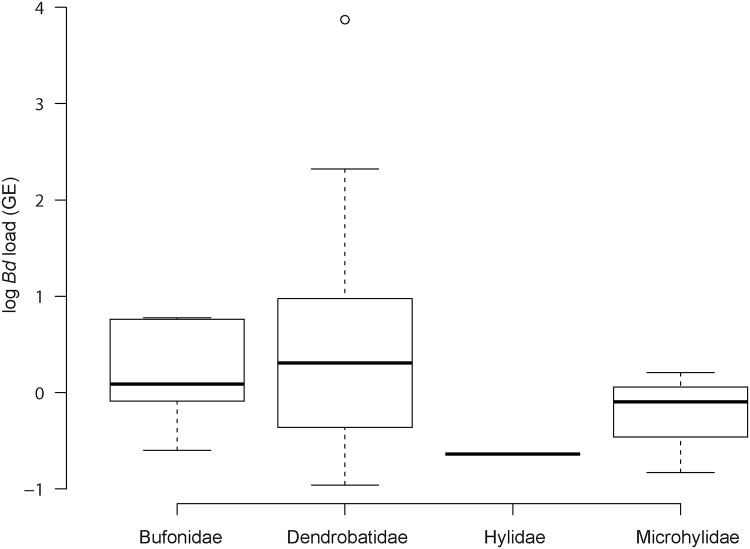
Variation of log (*Bd* load) among families.

In 2011–2012, prevalence of *Bd* across species varied from 0% to 43.3% with the highest prevalence found for *Allobates femoralis* (43.3%), followed by *Ranitomeya amazonica* (13.3%), *Anomaloglossus baeobatrachus* (12.1%), and *Dendrobates tinctorius* (9.3%; Table 3). Species in the Dendrobatidae family showed the highest prevalence (12.4%) as compared to other families (Wilcoxon test, *W* = 41, *p* = 0.04), mainly driven by the high prevalence found in *Allobates femoralis* (Table 3). We did not detect a significant difference of *Bd* load across the four families (Dendrobatidae, Bufonidae, Hylidae, Microhylidae; Kruskal Wallis test, df = 3, *K* = 2.7, *p* = 0.45, Fig 2). Nonetheless, high *Bd* loads (greater than 50 GE) were found only for *D*. *tinctorius* (reaching up to 7420 GE) and *A*. *baeobatrachus* individuals (S1 Table). All but one population of *D*. *tinctorius* were *Bd*-positive with a prevalence ranging from 4.4% to 16.0% (Table 3). For *A*. *baeobatrachus*, two sites out of four were positive with high prevalence (25.7% and 10.7%; Table 3).

**Table 3:** *Bd* prevalence across species and sites.

	(A)					(B)				(C)				(D)	Cross-species prevalence
	*Dt*	*Af*	*Ab*	*A aff d*	*Ra*	*Rm*	*Rc*	*At*	*A sp*.	*Dm*	*Dl*	*Hp*	*Sb*	*Cs*	
**ADNG (N = 79)**	-	-	0% (18)	-	-	0% (11)	-	-	-	**3.3% (30)**	0% (10)	0% (10)	-	-	**1%**
Trinité (N = 30)	-	-	-	-	-	0% (31)	-	-	-	-	-	-	-	-	0%
Saint Elie (N = 55)	-	-	0% (18)	0% (20)	-	0% (8)	0% (9)	-	-	-	-	-	-	-	0%
**Montagne des singes (N = 56)**	-	-	**25.7% (35)**	-	-	0% (5)	0% (16)	-	-	-	-	-	-	-	**16%**
**Matoury (N = 30)**	-	-	-	-	-	-	-	**13.3% (30)**	-	-	-	-	-	-	**13%**
**Favard (N = 50)**	**16% (50)**	-	-	-	-	-	-	-	-	-	-	-	-	-	**16%**
**Trésor (N = 70)**	**8.3% (24)**	-	-	-	-	0% (2)	0% (7)	0% (7)	-	-	-	-	-	**10% (30)**	**7%**
**Inselberg (N = 99)**	**14% (43)**	-	-	-	-	0% (21)	0% (3)	0% (32)	-	-	-	-	-	-	**6%**
**Pararé (N = 175)**	**4.4% (91)**	**43.3% (30)**	-	-	-	0% (23)	**25% (4)**	**7.4% (27)**	-	-	-	-	-	-	**11%**
**Saut Maripa (N = 78)**	0% (7)	-	**10.7% (28)**	-	**13.3% (15)**	0% (3)	0% (14)	-	0% (11)	-	-	-	-	-	**6%**
Saint-George (N = 24)	-	-	-	-	-	-	-	-	0% (5)	-	0% (7)	-	0% (12)	-	0%
	**9.3% (215)**	**43.3% (30)**	**12.1% (99)**	0% (20)	**13.3% (15)**	0% (104)	**1.9% (53)**	**6.3% (96)**	0% (16)	**3.3% (30)**	0% (17)	0% (10)	0% (12)	**10% (30)**	
	12.4% (379)					2.6% (269)				1.5% (69)				-	

The number of individuals sampled is indicated in brackets for (A) Dendrobatidae (*Dt—Dendrobates tinctorius*, *Af—Allobates femoralis*, *Ab—Anomaloglossus baeobatrachus*, *A aff*. *d—Anomaloglossus aff*. *degranvillei*, *Ra—Ranitomeya amazonica*), (B) Bufonidae (*Rm—Rhinella margaritifera*, *Rc—R*. *castaneotica*, *At—Atelopus flavescens*, *A sp*.*—Amazophrynella sp.)*, (C) Hylidae (*Dm—Dendropsophus minutus*, *Dl—D*. *leucophyllatus*, *Hp—Hypsiboas punctatus*, *Sb—Scinax boesmanii)* and (D) Microhylidae (*Cs*—*Chiasmocleis shudikarensis*). Infected sites and species are highlighted in bold.

**Fig 3:**
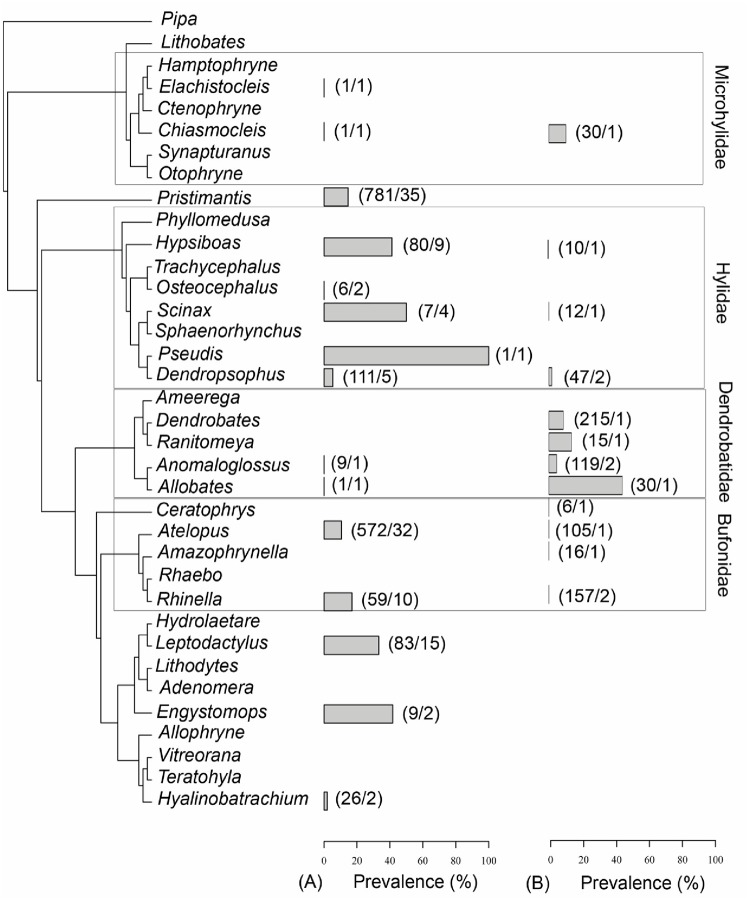
Measure of *Bd* prevalence for anuran genera. (A) Mean prevalence for the genus as reported in *Bd-*maps and (B) Mean prevalence for the genus measured in this study. Values in bracket indicate the total number of individuals sampled and the number of species sampled for the genus (Number of individuals/Number of species). The phylogenetic tree (topology) is based on Pyron et al. (2011) adapted according to Fouquet et al. (2013).

In the meta-analysis, only two species of Dendrobatidae had already been tested in the wild and only with small sample sizes (Fig 3). Among the genera that had not been tested in this study, two had been intensively tested for *Bd* presence in other countries (*Pristimantis*, Craugastoridae with 35 species and 781 individuals and *Leptodactylus*, Leptodactylidae with 15 species and 83 individuals). Mean prevalence in these genera were high (greater than 15%).

Of the 346 samples from 2009, 10 were *Bd*-positive (2.9%, S1 Table). In 2011–2012, 58 out of 747 samples (7.7%, S1 Table) were *Bd*-positive. Two significant increases in *Bd* prevalence were recorded between 2009 and 2011–2012 (Table 4): one for *A*. *femoralis* at the Pararé site (Fisher test, *p* = 0.02, Table 4) and one for *D*. *tinctorius* at the Favard site (Fisher test, *p* = 0.009, Table 4). All other changes in *Bd* prevalence were not significant (Fisher test, *p*>0.05).

**Table 4:** *Bd* prevalence in 2009 and 2011.

Site	Year	*Dendrobates tinctorius*	*Allobates femoralis*	*Rhinella margaritifera*	*Atelopus flavescens*
Favard	2009	**4.6% (152)**	-	-	-
	2011	**16% (50)**	-	-	-
Inselberg	2009	0% (7)	-	0% (27)	0% (7)
	2011	14% (43)	-	0% (21)	0% (32)
Pararé	2009	17% (18)	**0% (10)**	-	0% (21)
	2011	4.4% (91)	**43.3% (30)**	-	7.4% (27)
Trésor	2009	0% (20)	-	0% (22)	-
	2011	8.3% (24)	-	0% (2)	-
Matoury	2009	-	-	-	0% (22)
	2011	-	-	-	13.3% (30)

The number of individuals sampled is indicated in brackets. Significant changes in *Bd* prevalence (Fisher test, *p*<0.05) are highlighted in bold.

**Fig 4:**
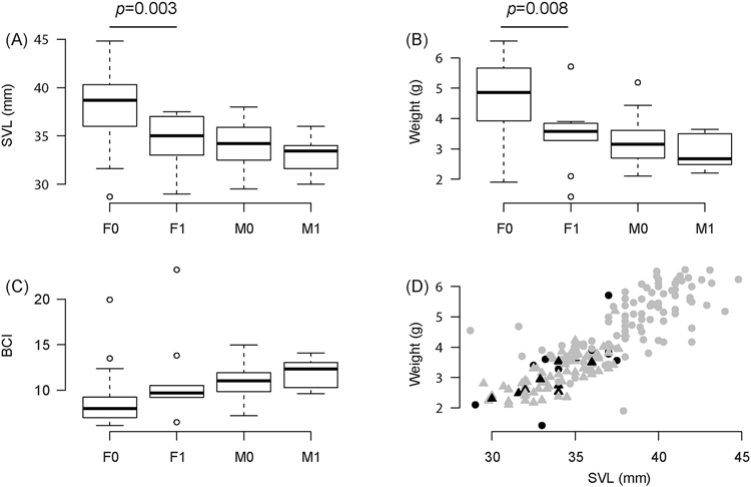
Effect of infection on SVL, weight and BCI for *D*. *tinctorius*. Distribution of (A) Snout Vent Length—SVL, (B) weight and (C) Body Condition Index (BCI) defined as the residuals of the regression between SVL and cube root of weight for *Dendrobates tinctorius* individuals for uninfected (F0; N = 107) and infected (F1; N = 9) females and for uninfected (M0; N = 107) and infected (M1; N = 11) males. Significant p-values of Wilcoxon tests are indicated in the figure. (D) Relationship between SVL (mm) and the cubic root of weight with infected individuals in black and uninfected individuals in grey. Males are indicated by triangles and females by points.

For *D*. *tinctorius*, we found that infected females tended to be significantly smaller (Wilcoxon test, W = 751, *p*<0.001) and thinner (Wilcoxon test, W = 739, *p* = 0.002) than uninfected females (N = 9 infected and 107 uninfected females; Fig 4). We could not observe a significant difference for males (SVL: W = 453, *p* = 0.27; Weight: W = 421, *p* = 0.35; N = 11 infected and 107 uninfected males). BCI did not significantly differ between infected and uninfected individuals of either sex (Male: W = 398, *p* = 0.97; Female: W = 560, *p* = 0.42; Fig 4).

## Discussion

Here, we report the distribution of the amphibian pathogen *Batrachochytrium dendrobatidis* in the amphibian rich region of French Guiana. We found that *Bd* fungus was widely distributed in French Guiana and among amphibian hosts. Indeed, eight out of the 11 sampled sites were found to be *Bd*-positive for at least one species at elevations ranging from 30 to 200 m a.s.l. The widespread distribution of *Bd* discovered here was unexpected in light of the published literature [20,21] and has implications for the protection of several species endemic of the Guiana shield. Our results confirm that *Bd* is not necessarily restricted to high-elevation tropical regions [43] but may infect species even in lowland tropics [15,16,25]. Such a finding is critical for a better understanding of the *Bd* distribution and its ecological niche in the tropics, which might markedly differ to what has been reported for other regions [44].

We found a difference in prevalence between Dendrobatidae, Bufonidae and Hylidae species, with Dendrobatidae species displaying the highest *Bd*-prevalence. Such differences among species have been previously reported but it remains unclear whether this can be explained by phylogenetic conservatism in *Bd*-resistance or by differences in breeding habitats [7,29]. In Australia, species associated with streams or permanent water bodies were more threatened than species with terrestrial reproductive systems [28,45] suggesting that prolonged stay of tadpoles in water may increase infection probability. Nonetheless, such effect can be mitigated by the presence of zooplankton in the reproduction sites [46,47]. In other localities as Colombia, species with water-independent habits have been shown to be those with the highest *Bd* prevalence [15]. In our study, Dendrobatidae species with terrestrial habits and reproducing in phytotelms or via direct development [48] displayed highest *Bd* prevalence. Published data on *Bd* prevalence for Dendrobatidae are scarce and the prevalence values determined here show that Dendrobatidae species may be more susceptible to *Bd* infection than previously thought. In this cross-site analysis, locality can be a confounding factor, but when analyzing sites separately, Dendrobatidae species always displayed the highest *Bd* prevalence.

For future *Bd* surveillance, it is also of importance to determine sentinel species to focus efforts on the most susceptible species and to detect the presence of *Bd* unambiguously. D*endrobates tinctorius*, *Allobates femoralis* and *Anomaloglossus baeobatrachus* displayed the highest *Bd* prevalence in this study and are locally abundant [49]. Moreover, we observed an increase of *Bd* prevalence in two species (*D*. *tinctorius* and *A*. *femoralis*) within a 3-year period. Those species may therefore be good sentinel species for *Bd* monitoring in French Guiana and the Guiana shield. In addition to the species tested in this study, other families (especially Leptodactylidae and Craugastoridae) may also display high *Bd* prevalence as suggested by analysis of published data. For *Bd* surveillance in French Guiana and the larger Guiana Shield, we therefore propose to focus efforts on *D*. *tinctorius*, *A*. *femoralis* and *A*. *baeobatrachus*, but suggest collecting Leptodactylidae and Craugastoridae species were in sympatry. The use of a standard protocol in *Bd* surveillance will enable long-term comparisons as done elsewhere in the world [50]. Such an approach would be beneficial in understanding the distribution pattern and the impact of *Bd* in amphibian rich countries like Madagascar in which *Bd* was recently proven to occur [51].

Our morphometric analysis for *D*. *tinctorius* suggests that infected females were on average smaller and thinner than non-infected ones. We did not find a similar relationship for males due to either a too small sample size or to sex-related differences in *Bd* susceptibility. In *D*. *tinctorius*, previous experiments in captive-bred individuals showed that older (and therefore larger) frogs tended to display an increased resistance to chytridiomycosis [52] and similar findings have been evidenced in other frog species [28]. Another explanation could be that *Bd* induces developmental stress that limits growth, a pattern evidenced in several species such as *Bufo fowleri* and *Hyla chrysoscelis* [53]. Our data are insufficient to definitively conclude that *Bd* infection impacts on the body condition or development of *D*. *tinctorius* and future studies, including experimental designs, should be conducted to assess the potential impact of *Bd* infection on *D*. *tinctorius* and other Dendrobatidae species. *Allobates femoralis* showed a high prevalence of *Bd*, yet the *A femoralis* populations at Nouragues have been intensively studied over the past two decades [54] with no evidence for a population decline over this period.

The impact of *Bd* for wild amphibian population in French Guiana remains unknown as no mortality events in association with *Bd* infection have been documented to date. High predation pressure and short degradation times in tropical forest ecosystems may impact the detectability of sick, dying or dead *Bd* positive individuals. We observed an increase of *Bd* prevalence in two species (*D*. *tinctorius* and *A*. *femoralis*) within a 3-year period suggesting that *Bd* may have recently established in French Guiana and that is now spreading. Even if some *Bd*-infected frog populations have shown no evidence of decline [11,55], other species underwent a rapid decline following infection after a prevalence threshold had been achieved [5,6]. Such difference in coexistence can be attributed to the context-dependent nature of susceptibility to a disease [44], the colonization success of *Bd* and zoospore density in a habitat [47,56], but the *Bd* genotype also has an important epidemiological determinant [27]. If the *Bd* strain present in French Guiana falls within the *Bd*-GPL lineage [27], several of the most iconic amphibian species of the Guiana Shield may be at serious risk of regional extinctions. Hence, a most pressing question is to understand whether the presence of *Bd* in French Guiana is also related to a high pathogenicity or if environmental determinants explain the observed distribution pattern.

## Supporting Information

S1 Table *Bd* load for positive samples. SP indicates single positive (considered as negative for the analysis) and DP double positive. Only samples labeled DP with a *Bd* load greater than 0.1 GE were considered as positive and are highlighted in bold.
(XLS)
